# Does Dysmenorrhea Affect Clinical Features and Long-Term Surgical Outcomes of Patients With Ovarian Endometriosis? A 12-Year Retrospective Observational Cohort Study

**DOI:** 10.3389/fmed.2022.905688

**Published:** 2022-06-16

**Authors:** Yushi Wu, Xiaoyan Li, Yi Dai, Jinghua Shi, Zhiyue Gu, Jing Zhang, Chenyu Zhang, Hailan Yan, Jinhua Leng

**Affiliations:** National Clinical Research Center for Obstetrics and Gynecologic Diseases, Department of Obstetrics and Gynecology, Peking Union Medical College Hospital, Chinese Academy of Medical Science and Peking Union Medical College, Beijing, China

**Keywords:** endometriosis, dysmenorrhea, pregnancy rate, laparoscopic cystectomy, recurrence rate, postoperative outcomes

## Abstract

**Background:**

To examine and compare the differences in clinical characteristics and long-term postoperative outcomes of ovarian endometriomas (OMA) patients with and without dysmenorrhea, including data from at least 8 years of postoperative follow-up examinations.

**Methods:**

Retrospective analysis of 334 OMA patients, including their demographic and clinical data. Long-term follow-up record was also collected. All laparoscopic cystectomy procedures were performed by the same surgeon at Peking Union Medical College Hospital between January 2009 and April 2013. Patients were divided into the dysmenorrhea and non-dysmenorrhea groups to perform the analysis of their preoperative characteristics, relevant surgical findings, and postoperative outcomes at the follow-up.

**Results:**

Out of 334 OMA patients, 257 (76.9%) patients were allocated to the dysmenorrhea group, while the rest 77 (23.1%) patients were included in the non-dysmenorrhea group. Compared with the dysmenorrhea group, the non-dysmenorrhea group exhibited a reduced proportion of chronic pelvic pain (CPP) (*P* = 0.003), dyspareunia (*P* < 0.001), tenesmus (*P* < 0.001), concurrency of deep infiltrating endometriosis (DIE) (*P* < 0.001), and adenomyosis (*P* = 0.032). Preoperative infertility was significantly higher in the dysmenorrhea group (*P* = 0.001). The mean operating time in the dysmenorrhea vs. the non-dysmenorrhea group was 68.0 vs. 56.0 min (*P* < 0.001). According to the revised American Fertility Society (rAFS) scoring system, the mean scores of the two groups were 52.1 vs. 44.6 (*P* = 0.033). During follow-up, the dysmenorrhea group showed a higher rate of disease relapse (*P* < 0.001). A minimum postoperative follow-up period of 8 years was required to evaluate the pregnancy outcomes. Successful pregnancies were identified in 97/257 (37.7%) cases in the dysmenorrhea group and 36/77(46.8%) cases in the non-dysmenorrhea group (*P* = 0.157), respectively. Though the dysmenorrhea group had a higher rate of postoperative infertility, differences were not significant between the two groups.

**Conclusions:**

Compared with the dysmenorrhea group, OMA patients without dysmenorrhea exhibited lower proportions of CPP, dyspareunia, tenesmus, lower concurrency of DIE and adenomyosis, shorter mean operating time, lower mean rAFS scores, and lower infertility rates. During the long-term follow-up, a lower recurrence rate was observed in the non-dysmenorrhea group. Regarding fertility outcomes, non-dysmenorrhea patients had a higher likelihood of successful pregnancy after surgery. Postoperative management needs to be evaluated separately according to dysmenorrhea pathology.

## Introduction

Endometriosis (EM), characterized by the abnormal or ectopic growth of stroma and endometrial glands outside the uterus (1), accounts for an estimated prevalence of 10–15% in females of reproductive age (2). Most EM lesions can be classified into 3 subtypes based on their locations and histological characteristics, including deep infiltrating endometriosis (DIE), cystic ovarian endometriosis/endometrioma (OMA), and superficial peritoneal endometriosis (SUP) (3). In some rare cases, endometriosis lesions can also appear in external pelvic sites such as skin and nasal cavity (4). Amongst EM lesion types, OMA is the most observed phenotype, accounting for 17–44% of EM cases. Laparoscopic cystectomy, the gold standard for OMA diagnosis, is also recommended as the first-line therapy (5–7).

Dysmenorrhea, together with infertility, constitutes the major clinical complication in OMA patients (8, 9). However, about 17–39% of OMA patients do not present any complaint of dysmenorrhea (10–12). Hence, whether dysmenorrhea symptoms would correlate with clinical characteristics and postoperative outcomes in this subset of OMA patients remain unclear.

## Methods

This study was performed in the Department of Obstetrics and Gynecology of the Peking Union Medical College Hospital (PUMCH), with prior approval from the Medical College Ethics Committee (JS-1722).

This study included OMA patients who received laparoscopic cystectomy at PUMCH between January 2009 and April 2013. All operations were performed by the same EM-specialized surgery team. The inclusion criteria were as follows: (1) patients were surgically treated in our hospital; (2) postoperative histopathological diagnosis of EM; (3) availability of complete clinical and pathological reports, and (4) participating patients were pre-menopausal. Cases, where patients had undergone similar surgeries in other hospitals were excluded from the analysis. Patients were also excluded if they were pregnant, had malignant gynecological diseases, or received any hormonal therapy (gonadotropin-releasing hormone analog, GnRHa; oral contraceptives, OCs, or levonorgestrel intrauterine system, mirena) within 3 months before the surgery.

The standard laparoscopic cystectomy surgery was executed as follows: (1) the extent of EM lesions and pelvic adhesions were identified by a thorough clinical inspection; (2) identifiable peritoneal implants were coagulated; (3) attempts were made to restore the normal anatomy as much as possible, and (4) ovarian cysts were carefully stripped off avoiding accidental damage to the surrounding healthy tissue. Regarding the surgery report of EM laparoscopy, the presence of lesions, anatomical location, the extent of subtle lesions and typical powder burns, pelvic adhesions, and the occurrence of deep infiltrating implants were recorded. The rAFS scoring system was applied to score patients along with the EM staging (13). DIE was diagnosed histologically if the lesion infiltrated >5 mm under the peritoneal surface. Visual analogique scale (VAS) was employed to evaluate the severity of pain symptom. VAS score≥3 was defined as dysmenorrhea.

According to these criteria, a total of 334 patients were pathologically diagnosed with OMA. Diagnosis of adenomyosis was confirmed when at least three or more of the following ultrasound features were present–globular-appearing uterus (regular enlarged uterus), the asymmetrical thickness of the anteroposterior wall of the myometrium, heterogeneous myometrial echotexture (presence of an indistinctly myometrial area with decreased or increased echogenicity), sub endometrial echogenic linear striations (radiate pattern of thin acoustic shadowing not arising from echogenic foci), sub endometrial myometrial cysts (round anechoic areas of 1–7 mm diameter), or poor definition of the endometrial-myometrial junction, as described elsewhere (14–18). Patients who received biopsy or myomectomy during the surgery were identified to have adenomyosis by the postoperative histopathology.

The size of the OMA was defined as the largest diameter of cysts measured by ultrasound examinations. The elevated serum CA-125 level was defined as >35 U/ml, per the clinical laboratory references at PUMCH. The possibility of infertility in women was diagnosed when the patient was unable to conceive for more than 1 year despite having a normal sex life without using any contraceptives, after ruling out any male etiological factors of infertility.

Clinical indications of eligible patients were retrospectively recorded and analyzed after thoroughly scrutinizing patients' medical records. The following demographic and medical information were retrieved: body mass index (BMI); age; parity; history of surgery; symptoms of dyschezia, dyspareunia, dysmenorrhea, dysuria; location of cysts; the size of EM lesion; serum CA-125 level; intraoperative blood loss; operating time, and rAFS stage. As the postoperative management, patients with fertility needs received a short-term GnRHa course of 3–6 injections or oral contraceptives. Furthermore, for those without fertility requirements, long-term therapy was recommended using Mirena (levonorgestrel intrauterine system) alone or a combination of GnRHa and Mirena. In the long-term therapy, it could be possible that some patients did not follow the exact time course of the treatment.

All patients were examined and followed up as per our internal protocol. A transvaginal ultrasound along with a standard gynecological examination was conducted for each patient before surgery, at 3, 6, and 12 months post-surgery, then yearly for the long-term follow-up. Menstrual fertility factors and pain symptoms were also evaluated. Patients were interviewed to reveal if they experienced any dysmenorrhea, dyspareunia, or pelvic pain symptoms during the follow-up. Pain recurrence was considered if it recurred after 3 months of surgery. Endometrioma was defined as the persistent presence of ovarian cysts with a thin wall (with a diameter of at least 2 cm), a homogenous low echogenic fluid content with scattered internal echoes regular margins, and persisted even after successive cycles of menstruation. Endometriosis recurrence was identified when pain, endometrioma, or both were noted at the follow-up ultrasound and physical examinations. The long-term postoperative follow-up information included postoperative medications, infertility and related symptoms, recurrence time, and imaging results.

Based on the diagnosis, patients were divided into either the dysmenorrhea or non- dysmenorrhea group to analyze and compare their demographic and clinical baseline characteristics, surgical observations, and postoperative outcomes during the follow-up examinations. Statistical analyses were performed using the IBM SPSS 25.0 software. The Kruskal-Wallis test was applied to analyze continuous variables, while categorical variables were analyzed using the chi-square test or Fisher's exact test. The Kaplan-Meier survival plots were constructed to measure recurrence rates. All statistical tests were two-sided, and *P* < 0.05 was set to identify the statistical significance of the result.

## Results

Out of 334 OMA patients participating in this study, 257 (76.9%) patients were included in the dysmenorrhea, and the remaining 77(23.1%) patients were in the non-dysmenorrhea group. Comparisons of clinical features and demographic features between these two groups are shown in Table 1.

**Table 1:** Comparison of demographic and clinical characteristics.

**Characteristic/group**	**Non-dysmenorrhea**	**Dysmenorrhea**	***P*-value**
Number (%)	77(23.1%)	257 (76.9%)	-
Mean age, y	32.69 ± 5.50	32.74 ± 5.23	0.941
BMI(Kg/m^2^)	21.01 ± 2.71	21.15 ± 2.60	0.696
Gravidity	1.09 ± 1.49	0.96 ± 1.21	0.433
Parity	0.33 ± 0.50	0.33 ± 0.50	0.958
CPP (%)	4 (5.19%)	50 (19.53%)	0.003
Dyspareunia (%)	5 (6.49%)	72 (28.12%)	<0.001
Tenesmus (%)	7 (9.09%)	75 (29.30%)	<0.001
Dyschizia (%)	2 (2.60%)	23 (8.98%)	0.062
CA-125, U/ml	81.92 ± 107.67	101.80 ± 219.75	0.454
Elevated CA-125 (%)	51 (68.00%)	175 (73.22%)	0.380
Infertility			0.010
No	70 (90.91%)	200 (77.82%)	
Infertility	7 (9.09%)	57 (22.18%)	
Size of OMA, cm			-
Left	5.57 ± 2.19	5.32 ± 2.07	0.424
Right	5.67 ± 1.68	5.22 ± 1.96	0.115
Leiomyoma (%)	14 (18.18%)	46 (18.04%)	0.977
Adenomyosis (%)	22 (28.57%)	108 (42.19%)	0.032

Between the dysmenorrhea and non-dysmenorrhea groups, the mean age, mean gravidity, and mean parity were 32.74 vs. 32.69 years (*P* = 0.941), 0.96 ± 1.21 vs. 1.09 ± 1.49 (*P* = 0.433), and 0.33 ± 0.50 vs. 0.33 ± 0.50 (*P* = 0.958), respectively. The CPP symptom was diagnosed in 50 (19.53%) vs. 4 (5.19%) cases in the dysmenorrhea vs. non-dysmenorrhea group (*P* = 0.003). Menstrual tenesmus was reported in 75 (29.30%) dysmenorrhea vs. 7 (9.09%) non-dysmenorrhea patients (*P* < 0.001). Dyspareunia was observed in 72 (28.12%) vs. 5 (6.49%) cases between the groups (*P* < 0.001). Dyschizia was complained in 23 (8.98%) dysmenorrhea vs. 2 (2.60%) non-dysmenorrhea patients (*P* = 0.062). The mean levels of CA-125 were found 101.80 vs. 81.92 U/ml (*P* = 0.454) in these groups, while elevated levels of CA-125 were detected in 175 (73.22%) vs. 51 (68.00%) patients (*P* = 0.380) in the dysmenorrhea vs. non-dysmenorrhea groups. Moreover, 108 (42.19%) vs. 22 (28.57%) cases were clinically diagnosed with adenomyosis in the dysmenorrhea vs. non-dysmenorrhea group (*P* = 0.032). The pre-surgery infertility was diagnosed in 57 (22.18%) vs. 7 (9.09%) cases in the dysmenorrhea vs. non-dysmenorrhea group (*P* = 0.010).

All patients received laparoscopic cystectomy, and the diagnosis of OMA was histopathologically determined. The pelvic cavity was carefully explored to remove or coagulate any visible peritoneal implants. DIE lesions were radically removed during the procedure. Intraoperative situations are listed in Table 2.

**Table 2:** Surgical situations.

**Characteristic/group**	**Non-dysmenorrhea**	**Dysmenorrhea**	***P*-value**
Operating time, min	56.0 ± 18.0	68.0 ± 21.3	<0.001
Mean bleeding volume, ml	39.7 ± 25.9	54.3 ± 57.7	0.032
DIE (%)	28 (36.4%)	150 (58.4%)	<0.001
Closed Douglas pouch (%)	43 (55.8%)	198 (77.0%)	<0.001
Multilocular (%)			-
Left	33 (70.2%)	118 (83.1%)	0.056
Right	36 (66.7%)	122 (73.5%)	0.333
rAFS score	44.6 ± 25.1	52.1 ± 25.2	0.033
Stage (%)			0.095
I	1 (1.3%)	0 (0.0%)	
II	2 (2.6%)	8 (3.1%)	
III	35 (45.5%)	90 (35.0%)	
IV	39 (50.6%)	159 (61.9%)	
Pelvic Endometriosis (%)			0.002
No	20(30.8)	33(19.0%)	
Peritoneal EM	29(44.6)	51(29.3)	
DIE	16(24.6)	81(46.6)	
Peritoneal EM+DIE	0	9(3.8)	
SUP (%)			0.857
Single	9 (26.47%)	25 (28.09%)	
Multiple	25 (73.53%)	64 (71.91%)	
SUP position (%)			
Broad ligament	5 (14.71%)	16 (17.98%)	0.666
Uterosacral ligament	22 (64.71%)	64 (71.91%)	0.436
Bladder peritoneal reflex	3 (8.82%)	11 (12.36%)	0.581
Ureter	4 (11.76%)	4 (4.49%)	0.144
Rectum	4 (11.76%)	7 (7.87%)	0.498
Uterus	5 (14.71%)	13 (14.61%)	0.989

Between the dysmenorrhea and non-dysmenorrhea groups, the mean surgery time was 68.0 vs. 56.0 min (*P* < 0.001), and the mean bleeding volume was 39.7 vs. 54.3 ml (*P* = 0.032). No significant differences were noticed between the two groups (*P* > 0.05) in the mean size of cysts and their laterality, as well as the percentage of multilocular cysts. During laparoscopy, 150 (58.4%) vs. 28 (36.4%) cases were diagnosed with the DIE in the dysmenorrhea vs. non-dysmenorrhea group (*P* < 0.001). Peritoneal endometriosis was observed in 123 patients. The position and invasion quantity of SUP revealed no significant differences among groups. The mean rAFS scores were 52.1 and 44.6 (*P* < 0.033) in the dysmenorrhea and non-dysmenorrhea groups, without any significant differences in stages (*P* = 0.095).

We examined the concurrency of pelvic EM in OMA patients, excluding the influence of adenomyosis. Subgroup comparison revealed concurrent DIE had a strong effect on dysmenorrhea (Table 3).

**Table 3:** Concurrent pelvic endometriosis conditions.

**Characteristic/group**	**Non-dysmenorrhea**	**Dysmenorrhea**	***P*-value**
Pelvic EM (%)			
No	20(40.8)	33(39.3)	0.862
Peritoneal EM	29(49.2)	51(61.7)	
No	20(55.6)	33(28.9)	0.004
DIE	16(44.4)	81(71.1)	
No	20(100)	33(78.6)	0.025
Peritoneal EM+DIE	0	9(21.4)	
Peritoneal EM	29(64.4)	51(38.6)	0.003
DIE	16(35.6)	81(61.4)	
Peritoneal EM	29(100)	51(85)	0.028
Peritoneal EM+DIE	0	9(15)	
DIE	16(100)	81(90)	0.186
Peritoneal EM+DIE	0	9(10)	

For the whole study group, all patients had a minimum of 96 months of outpatient follow-up. No significant difference was seen in the follow-up time between the dysmenorrhea and non-dysmenorrhea groups (120.47 vs. 120.64 months; median: 119.45 months; *P* = 0.929). Postoperative outcomes are shown in Table 4.

**Table 4:** Postoperative outcomes.

**Characteristic/group**	**Non-dysmenorrhea**	**Dysmenorrhea**	***P*-value**
Follow-up time, m	120.64 ± 15.29	120.47 ± 14.43	0.929
Postoperative therapy (%)			0.001
No treatment	6 (7.8%)	4 (1.6%)*	
GnRHa	49 (63.6%)	187 (72.7%)	
OCs	8(10.4%)	4(1.6%)*	
Mirena	2(2.6%)	11(4.3%)	
GnRHa+Mirena	12(15.6%)	51(19.8%)	
Treatment regimen			0.40
Short-term treatment	57(80.3%)	191(75.5%)	
Long-term treatment	14(19.7%)	62(24.5%)	
Recurrence (%)	3 (3.9%)	62 (24.1%)	<0.001
Type of recurrence (%)			<0.001
No	74 (96.1%)	195 (75.9%)	
Pain	0 (0.0%)	34 (13.2%)	
Endometrioma	3 (3.9%)	18 (7.0%)	
Both	0 (0.0%)	10 (3.9%)	
Pregnancy after surgery (%)			0.051
Spontaneous Pregnant	33(42.9%)	75(29.1%)*	
Artificial Pregnant	3(3.9%)	22(8.6%)	
Failed	41 (53.2%)	160 (62.3%)	
Pregnancy outcome in infertility patients(%)			0.52
Failed	3(42.9%)	24(42.1%)	
Spontaneous	4(57.1%)	23(40.4%)	
Artificial	0	10(17.5%)	

*Indicates the proportion of two groups are significantly different from each other (p <0.05).

In this study, 324 patients received postoperative medical therapy, including 253 (253/257, 98.4%) dysmenorrhea patients and 71 (71/77, 92.2%) non-dysmenorrhea patients (*P* = 0.005). The difference between treatment regimens in the two groups was non-significant (*P* = 0.40). GnRHa was the most commonly used postoperative treatment in both two groups. No significant differences were noticed between the two groups in the proportion of postoperative GnRHa administration. A higher proportion of non-dysmenorrhea patients had no postoperative medical treatment (7.8% vs. 1.6%).

Successful pregnancy cases were identified in 97/257 (37.7%) patients in the dysmenorrhea group vs. 36/77 (46.8%) patients in the non-dysmenorrhea group (*P* = 0.157) after at least 8 years of follow-up. Successful pregnancy and live births, including both *in vitro* fertilization (IVF-ET) and spontaneous pregnancy, were observed in 93/97 (95.9%) vs. 36/36 (100%) patients comparing the dysmenorrhea vs. non-dysmenorrhea groups, while spontaneous abortions were reported in the remaining patients. In all 64 patients who were preoperatively diagnosed with infertility, 37 patients got pregnant after surgery. Twenty-seven patients achieved spontaneous pregnancy while 10 patients undergone ART. In the non-dysmenorrhea group, 4 patients (57.1%) had spontaneous pregnancy after surgery, and 3 patients (42.9%) remained infertile. In dysmenorrhea group, 23 patients (40.4%) had spontaneous pregnancy, 10 patients (17.5%) conceived artificially, 24(42.1%) patients remain infertile.

The recurrence rate of the dysmenorrhea group was significantly higher than that of the non-dysmenorrhea group (24.1% vs. 3.9%, *P* < 0.001) after completion of the follow-up. Moreover, the 5-year cumulative recurrence rate was 17.9% vs. 3.9% in these two groups (*P* = 0.00014).

## Discussion

EM is usually diagnosed as an estrogen-dependent benign medical condition found in 10–15% of women in their reproductive ages. OMA lesions are mostly detected among all EMs. Laparoscopic cystectomy is regarded as the first line of treatment in EM management (19). Here, we investigated the clinical characteristics of EM patients with or without dysmenorrhea, involving a regular follow-up for 8–12 years, which showed women without dysmenorrhea had a lower recurrence rate.

The EM symptoms widely vary among individual subjects and disease stages (20), including pain symptoms (dysmenorrhea, dyspareunia, chronic pelvic pain), and infertility. Pain is the most important symptom in patients with EM, but underlying mechanisms remain unclear. Several pathophysiological hypotheses had been proposed in this regard, such as hormone-regulated neuro-vascular pathology (21); estrogen-dependent chronic inflammation in the presence of ectopic endometrial tissue invasions (22, 23); increased prostaglandin level, and lowered threshold of pain perception due to sensitization of nerve endings (24, 25); compression and infiltration of adjacent nerves (26), and relatively higher density of nerve fibers due to increased expressions of nerve growth factors, angiogenesis, and pathological changes leading to nerve innervation into the uterus, may contribute to the central and cross-organ sensitizations (27–30). Despite dysmenorrhea being a common clinical symptom in EM patients, we noticed that some patients diagnosed with EM did not present with dysmenorrhea. Clinical features and long-term postoperative outcomes of non-dysmenorrhea patients have not been investigated to date.

In this study, we found that 23.1% of OMA patients manifested no dysmenorrhea, which was in agreement with previous findings that 17–39% of OMA patients could be non-dysmenorrhea (10–12). According to our results, baseline characteristics such as age, BMI, parity, and gravidity showed no difference between the two groups. Patients in the dysmenorrhea group were associated with relatively higher proportions of CPP, dyspareunia, and tenesmus. The concurrency rates of adenomyosis and DIE were also higher in the dysmenorrhea group. There is accumulating evidence indicating that the presence of adenomyosis and DIE are strongly associated with dysmenorrhea (31, 32). Endometriosis, in particular DIE, was also proved to aggravate tenesmus by causing pelvic floor dysfunctions (33). It might further suggest that an increased number of patients in the dysmenorrhea group had DIE and adenomyosis, and this also explains the reason for frequently observed CPP, dyspareunia, and tenesmus symptoms in this group.

Several studies have reported that DIE patients present more severe sexual dysfunctions and dyspareunia (34), and the depth and number of infiltration are associated with the dysmenorrhea severity (32, 35, 36). We initially analyzed the location and size of the concurrent pelvic EM, including SUP and DIE. Concurrent DIE was associated with dysmenorrhea. There was no significant difference in the invasive position and number of peritoneal EM between the dysmenorrhea and non-dysmenorrhea groups. Subgroup analysis also revealed that DIE was the key factor determining the occurrence of dysmenorrhea.

Surgery is the primary treatment option for OMA patients. Our results showed that the mean operating time was significantly longer in the dysmenorrhea group. Also, the mean bleeding volume and mean rAFS score were significantly higher in the dysmenorrhea group than that in the non-dysmenorrhea group.

In this study, 324 patients received postoperative pharmacological therapy. In some centers, oral contraceptives or progesterone pills are first-line postoperative therapy to prevent endometriosis recurrence (37, 38). We noticed that GnRHa was the most commonly used postoperative treatment in our center for both patients with and without dysmenorrhea. Patients without dysmenorrhea had a higher proportion of no medical therapy after surgery. Despite the higher rate of postoperative observation, a lower recurrence rate was observed in non-dysmenorrhea patients during the long-term follow up. These results suggest that the risk of EM recurrence is affected not only by postoperative medication but also by the clinical features of the disease.

The possible reason of high recurrence rate in the dysmenorrhea group might also be the concurrence of peritoneal EM and DIE in this subset of patients. Previous research had revealed that patients who received radical surgery of rectosigmoid DIE lesions showed lower suspected recurrence rates than patients who accepted conservative surgery (39). DIE lesions are often difficult to remove completely during surgery, and invisible SUPs are almost impossible to remove by surgery, which might have contributed to higher postoperative recurrence rates in the dysmenorrhea group.

Importantly, patients in the dysmenorrhea group exhibited a higher rate of preoperative infertility compared with patients in the non-dysmenorrhea group. About one-third of women with EM suffer from infertility, which is approximately twice the rate in women without EM pathology (40). It has been suggested that the extent of disease progression and the degree of reduced spontaneous fertility in EM patients could be interrelated, but the strength of this association is not well-defined. In comparison to OMA and DIE, SUP lesions are more closely associated with infertility (41). In our study, patients in the dysmenorrhea group showed higher proportions of SUP and DIE lesions, along with higher rAFS scores, indicating the occurrence of more severe intraoperative pelvic adhesions in this group of patients. Severe pelvic adhesions may distort the dissection of fallopian tubes, thereby affecting the patient's pregnancy.

At least 8 years of the post-surgical follow-up period was required for patients to reveal the actual pregnancy outcomes. We found that non-dysmenorrhea patients had a higher postoperative pregnancy rate, especially spontaneous pregnancy rate, than dysmenorrhea patients. It suggested that non-dysmenorrhea patients had better long-term pregnancy outcomes. As previously analyzed, patients with dysmenorrhea are usually closely related to the presence of DIE and adenomyosis, and they usually have higher rAFS scores. These patients have more severe pelvic adhesions and a more common inflammatory environment in the pelvic cavity. The presence of adenomyosis also interferes with embryo implantation and endometrial receptivity, which further affects the patient's long-term pregnancy outcomes. Importantly, patients in the dysmenorrhea group exhibited a higher rate of preoperative infertility compared with patients in the non-dysmenorrhea group. It could also be explained by the higher proportion of pelvic adhesions and adenomyosis in the dysmenorrhea group. As for postoperative long-term pregnancy outcomes, over half of the patients with a preoperative diagnosis of infertility had pregnancy after surgical procedure. Although there was no significant difference, patients in the non-dysmenorrhea group demonstrated a higher proportion of spontaneous pregnancies than those with dysmenorrhea. Previous studies had revealed that endometrioma cystectomy may have benefits on the chances of spontaneous conception (42). Cystectomy for endometrioma has certain effect on ovarian reserve function. Nevertheless, these negative effects of endometriosis do not reflect on pregnancy and delivery rates, which remain fully comparable and overlap with other causes of infertility (43, 44). Our result is consistent with previous research and further illustrates the relationship between postoperative pregnancy outcomes and dysmenorrhea.

As far as we are aware, the major strength of our study is that compared with previous studies, our data were collected after a real long-term follow-up, which was 8–12 years. For our patients, all the surgeries were performed primarily by one surgeon, which can eliminate many inherent confounding factors like introducing selection bias. This study also has some limitations. As a retrospective study, the main limitations of our study were recall bias. An observational design means that causal relationships cannot be established between factors and outcomes. At the same time, as a retrospective study of patients with a single surgeon in a single-center, some results may not be generalizable to a larger population. Considering the limitations of this study, in future investigations, multiple prospective studies involving non-dysmenorrhea patients are warranted to develop predictive models for clinicians toward a better understanding of the treatment strategies and prognosis.

## Conclusions

In this study, we summarized the clinical characteristics, surgical findings, and postoperative outcomes of OMA patients without dysmenorrhea in a long-term follow-up. Compared with dysmenorrhea, non-dysmenorrhea was significantly correlated with a lower proportion of CPP, dyspareunia, tenesmus, lower concurrency of DIE, adenomyosis, shorter operating time, a lower rAFS score, and lower infertility rate.

Also, non-dysmenorrhea patients exhibited a significantly lower rate of recurrence during the long-term follow-ups. Therefore, gynecologists should pay special attention to the clinical features of OMA patients. Non-dysmenorrhea patients have milder surgical difficulties and lower postoperative recurrence rates. The postoperative treatments need to be discussed separately.

## Data Availability Statement

The raw data supporting the conclusions of this article will be made available by the authors, without undue reservation.

## Ethics Statement

The studies involving human participants were reviewed and approved by Peking Union Medical College Hospital Review Board, reference number: JS-1532. Written informed consent for participation was not required for this study in accordance with the national legislation and the institutional requirements.

## Author Contributions

Material preparation and data collection were completed by YW and XL. Data analysis was performed by YW. The first draft of the manuscript was written by YW and XL. All authors commented on previous versions of the manuscript, contributed to the study conception and design, and approved publication of the final version.

## Funding

This study was funded by the National Key R&D Program (Grant Number: 2017YFC1001200) and National Natural Science Foundation of China (82071628).

## Conflict of Interest

The authors declare that the research was conducted in the absence of any commercial or financial relationships that could be construed as a potential conflict of interest.

## Publisher's Note

All claims expressed in this article are solely those of the authors and do not necessarily represent those of their affiliated organizations, or those of the publisher, the editors and the reviewers. Any product that may be evaluated in this article, or claim that may be made by its manufacturer, is not guaranteed or endorsed by the publisher.
